# Modeling Enteropathy or Diarrhea with the Top Bacterial and Protozoal Pathogens: Differential Determinants of Outcomes

**DOI:** 10.1021/acsinfecdis.0c00831

**Published:** 2021-04-26

**Authors:** Richard L. Guerrant, David T. Bolick, Jonathan R. Swann

**Affiliations:** † Center for Global Health Equity, Division of Infectious Diseases and International Health, University of Virginia School of Medicine, Charlottesville, Virginia 22908, United States; ‡ School of Human Development and Health, Faculty of Medicine, University of Southampton, Southampton SO16 6YD, United Kingdom; § Department of Metabolism, Digestion, and Reproduction, Faculty of Medicine, Imperial College London, London SW7 2AZ, United Kingdom

**Keywords:** enteropathy, diarrhea, animal models, stunting, cognitive development, inflammation

## Abstract

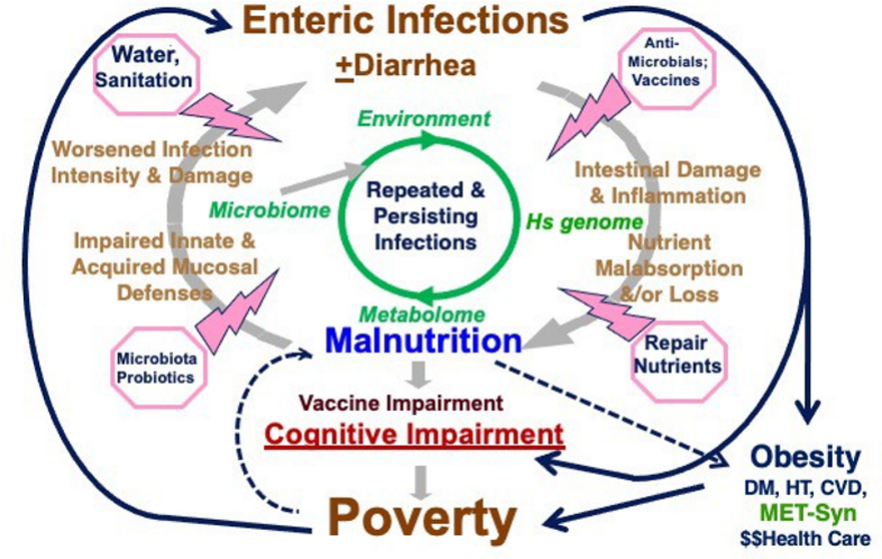

Developing effective therapeutics or preventive interventions for important health threats is greatly enhanced whenever accessible models can enable the assessment of clinically important outcomes. While no non-human model is ever perfect, inexpensive *in vivo* small animal models in such as mice are often of great help in assessing the relevant efficacy of potential interventions. In addition to acute diarrhea, the long-term growth and developmental effects of enteric infections, with or without overt diarrhea, are increasingly recognized. To address these diverse effects, inexpensive animal models are proving to be very helpful. Herein, we review the major clinical concerns with enteric parasitic and bacterial infections that are extremely common worldwide, especially in vulnerable young children living in impoverished areas, and the recently published murine models of these infections and their outcomes. We find that common dietary deficiencies seen in children in developing areas have striking effects on diarrhea and enteropathy outcomes in mice. However, these effects differ with different pathogens. Specifically, the effects of protein or zinc deficiency differ considerably with different major protozoal and bacterial pathogens, suggesting different pathogenetic pathways and intervention effects. The pathogens reviewed are the seven top parasitic and bacterial pathogens seen in children, namely, *Cryptosporidium*, *Giardia*, *Campylobacter*, *Shigella*, enterotoxigenic *Escherichia coli* (ETEC), enteroaggregative *E. coli* (EAEC), and enteropathogenic *E. coli* (EPEC).

## Introduction

Clinically relevant “disease” is classically seen as specific unhealthy outcomes for the “host” as a result of host and environmental determinants. On the host side, genetics are the obvious key (be it inherited or “epigenetic”), while the environment includes a wide range of “exposures” from physical characteristics (atmosphere, temperature, diet, antigens, toxins, *etc.*) to microbiologic (including endogenous microbiota, environmental milieu, or, for infectious diseases, exogenous “pathogens”). Understanding and ameliorating disease, or ideally preventing diseases or maintaining good “health”, requires an understanding of the causal relationships of the observed associations in clinical or field assessments. However, controlling relevant variables is key to experimental proof of causality but is often difficult or impossible to achieve in humans. Besides targeted, effective treatment (that is rarely sufficiently specific to be conclusive), experimental model systems have a critical role in establishing causal factors that impair health and are key to effective preventive or therapeutic interventions. Ideal “models” experimentally replicate the human conditions and their responses to interventions and range from *in silico* or *in vitro* to *in vivo* animal models. Likewise, defining a “disease” entity is critical to its recognition as well as to assessing preventive or treatment interventions. For example, proper surveillance to determine disease outbreaks first involve a careful “case definition” whether we seek to understand an Ebola outbreak or to better detect, prevent, and treat rheumatic fever or enteropathy.

This review is focused on the health impacts of common intestinal infections, especially in young, often undernourished children in impoverished settings worldwide. The obvious first enteric infectious “disease” that comes to mind is “diarrhea”. However, other, potentially even more impactful outcomes of intestinal infections now being recognized include “environmental enteropathy” (to distinguish it from immunologic enteropathies, as we currently understand inflammatory bowel diseases, Crohn’s Disease, and ulcerative colitis; although celiac disease may be considered environmental but nonmicrobial). We have proposed new terms for the growth impairment, cognitive decline, and metabolic syndrome that may follow early childhood enteric infections. “HAZdrop” refers to the growth impairment or decline in height-for-age Z [HAZ] scores in the first 2–3 years of life. “COGhit” refers to the impairment of cognitive development that can be attributed to enteric infections in early childhood. Such a cognitive impairment may also occur with enteric infections among the elderly, as well. “METsyn” refers to later life metabolic syndrome that can follow early life enteric infections.^1^ Although each of these outcomes, and the virulence traits of the pathogens themselves, are profoundly influenced by the dietary environment, they can be objectively measured for assessing interventions such as effective treatments, vaccines, or preventive measures.

Hence, it is both overt diarrhea as well as growth, weight gains, and measurable markers of enteropathy (histologic, barrier function, or inflammatory biomarkers) that we have focused on “modeling” in this review of our experience with murine models of enteric infections and their outcomes.

## Clinical/Field Data

### Diarrhea and Stunting Morbidities

Early childhood mortality from overt diarrhea remains troubling, killing nearly 500,000 children per year, or over 1000 children each day. In addition, a “silent” pandemic of moderate to severe stunting still affects an estimated 144 million children worldwide in the first two critically formative years of their lives, mostly in impoverished communities.^2,3^ This “silent pandemic” has potentially devastating consequences for children that do not die and may not be overtly symptomatic but who live through malnutrition and repeated or multiple enteric infections in early life. There has been a steady decline in the height-for-age Z (HAZ) scores of children in Asia, Africa, and Latin America over their first 2 years of life^4^ While the causes of this early childhood stunting in poor areas is complex, it likely relates to combinations of reduced food security and repeated or even common multiple enteric infections, resulting in damage to the intestine referred to as “environmental enteropathy” (EE) or environmental enteric dysfunction (EED). EE disrupts the absorptive and barrier functions of the gut, resulting in intestinal and systemic inflammation. The association of common intestinal infections in early childhood with impaired growth was seminally shown by Leonardo Mata, working with Nevin Scrimshaw, who correlated growth failure with repeated enteric and other infections that peak in the 4–24 month age window.^5^ They illustrated the sequence of repeated diarrhea and other illnesses being followed by declines in the growth curves of specific children in Santa Maria Cauque that “falls off” their expected growth trajectory when repeated enteric and other illnesses occur, often at weaning with its concomitant increase in crawling around in and consuming from contaminated environments. Remarkably, malnourished children who experience less diarrhea experience impressive “catch-up” growth, an effect that has been shown to be linearly ablated with progressively heavier diarrhea burdens seen in children in our studies in Northeast Brazil who were severely malnourished.^6^ Conversely, malnourished children experience both greater incidence and severity of diarrhea, completing a bidirectional vicious cycle of diarrhea and malnutrition.^7^

## Associations of Growth Impairment with Common Illnesses and Pathogens Associated with Intestinal or Systemic Inflammation

**Figure 1:**
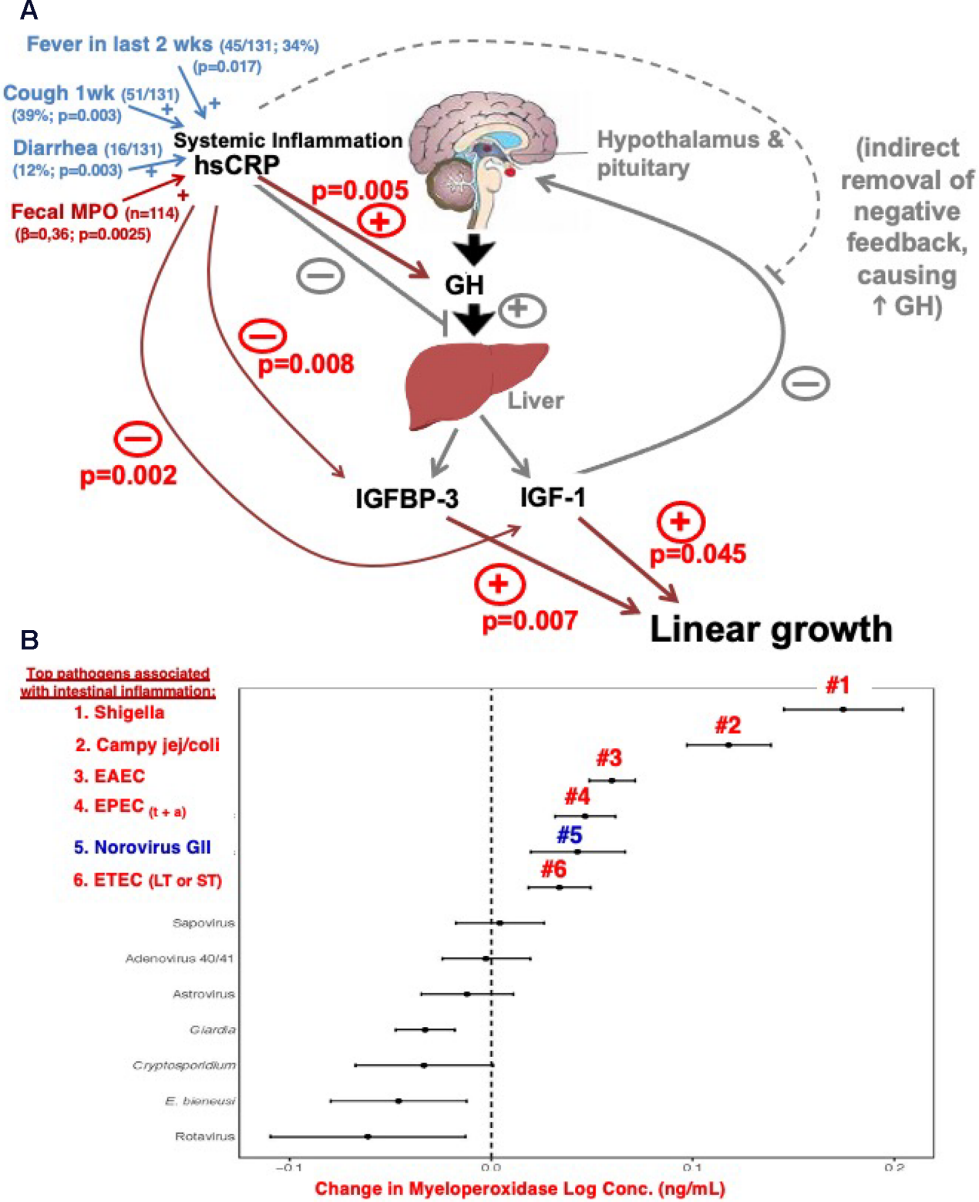
(A) Pathways involved in growth impairment that is significantly associated with the clinical signs of fever, cough, diarrhea (shown in blue), or intestinal inflammation (as assessed by fecal myeloperoxidase [MPO] shown in dark red) with systemic inflammation (hsCRP); of CRP with growth hormone signaling (GH, IGFBP-3, and IGF-1), and of IGFBP-3 and IGF-1 linear growth in young children in Northeast Brazil. The *p* values for each association are shown in red, along with + or – for positive or negative associations, respectively.^8,9^ (B) Associations of fecal MPO in 20,167 “silent” (asymptomatic) monthly specimens from young children in all eight MAL-ED cohorts by TAC, showing that *Shigella*, *Campylobacter*, EAEC (enteroaggregative *E. coli*), EPEC (enteropathogenic *E. coli*, both typical and atypical), group II norovirus (shown in blue), and ETEC (either LT- or ST-producing enterotoxigenic *E. coli*) are each significantly associated with this marker of intestinal inflammation (Platts-Mills, unpublished personal communication with permission, 2020). Analyses used a linear mixed effects model with outcome of log concentration of MPO, with covariates including pathogens, sex, age, age^2^, and random effects for site and individual. hsCRP = high sensitivity C-reactive protein; GH = growth hormone; IGFBP- 3 = insulin-like growth factor binding protein 3; IGF-1 = insulin-like growth factor 1.

The associations of common childhood illnesses with growth failure have continued to expand since Mata’s descriptions. In addition to the MAL-ED and GEMS studies discussed below, plausible mechanisms of local and systemic inflammation have been linked to impaired growth, as shown in Figure 1. Figure 1a summarizes both the significant associations of histories of recent fever, cough, or diarrhea, as well as fecal inflammation (from fecal myeloperoxidase, MPO, measurements) in a case-control study of malnourished children in Northeast Brazil with their systemic biomarkers of inflammation and growth signaling, including highly sensitive C-reacting protein (hsCRP), growth hormone (GH), IGFBP-3 and IGF-1, as well as likely pathways involved in these relationships.^8,9^ A recent clinical history of fever, cough, or diarrhea is each significantly associated with hsCRP (*p* = 0.017, 0.003, and 0.003, respectively) as is fecal MPO (*p* = 0.0025). The association of hsCRP with *increased* growth hormone (GH, *p* = 0.005) sounds initially counterintuitive. However, this is explained by the reduced hepatic responses to GH, IGFBP-3, and IGF-1 (*p* = 0.007 and 0.045, respectively), thus reducing their negative feedback on GH secretion.

Hence, the effects of intestinal and systemic inflammation on impaired hepatic responses to GH help explain the likely pathways involved in the growth impairment seen with common intestinal and other infections in early childhood.

Figure 1b further extends the importance of these findings and their potential relationship to “asymptomatic” intestinal infections (*i.e.*, without overt diarrhea), showing the significant associations of specific pathogens with growth failure. These data derive from TaqMan Array Cards (TAC PCR) for 29 different enteric pathogens in the stools of 20,167 “monthly” stool samples taken from over 1400 asymptomatic young children across all 8 sites in the MAL-ED studies (Platts-Mills, unpublished personal communication with permission).^10^ Quantitative molecular diagnostic methods to investigate the effect of specific enteric infections on linear growth in children in low-resource settings included longitudinal analysis of results from the MAL-ED cohort study.^11^ Impressively, the pathogens that were significantly associated with increased fecal MPO included (in order of highest to lower significant associations) *Shigella*, *Campylobacter*, enteroaggregative *E. coli* (EAEC), enteropathogenic *E. coli* (EPEC; both typical and “atypical”, *i.e.*, strains having *eae* without the *bfp* gene), and enterotoxigenic *E. coli* (ETEC; both LT-ETEC and ST-ETEC). Indeed, these are the top pathogens associated with impaired growth, all bacteria except group II noroviruses, either in this or in earlier studies.^12−15^

## Multicountry, Prospective, Field Cohort Studies of Frequency and Impact of Enteric Infections

**Figure 2:**
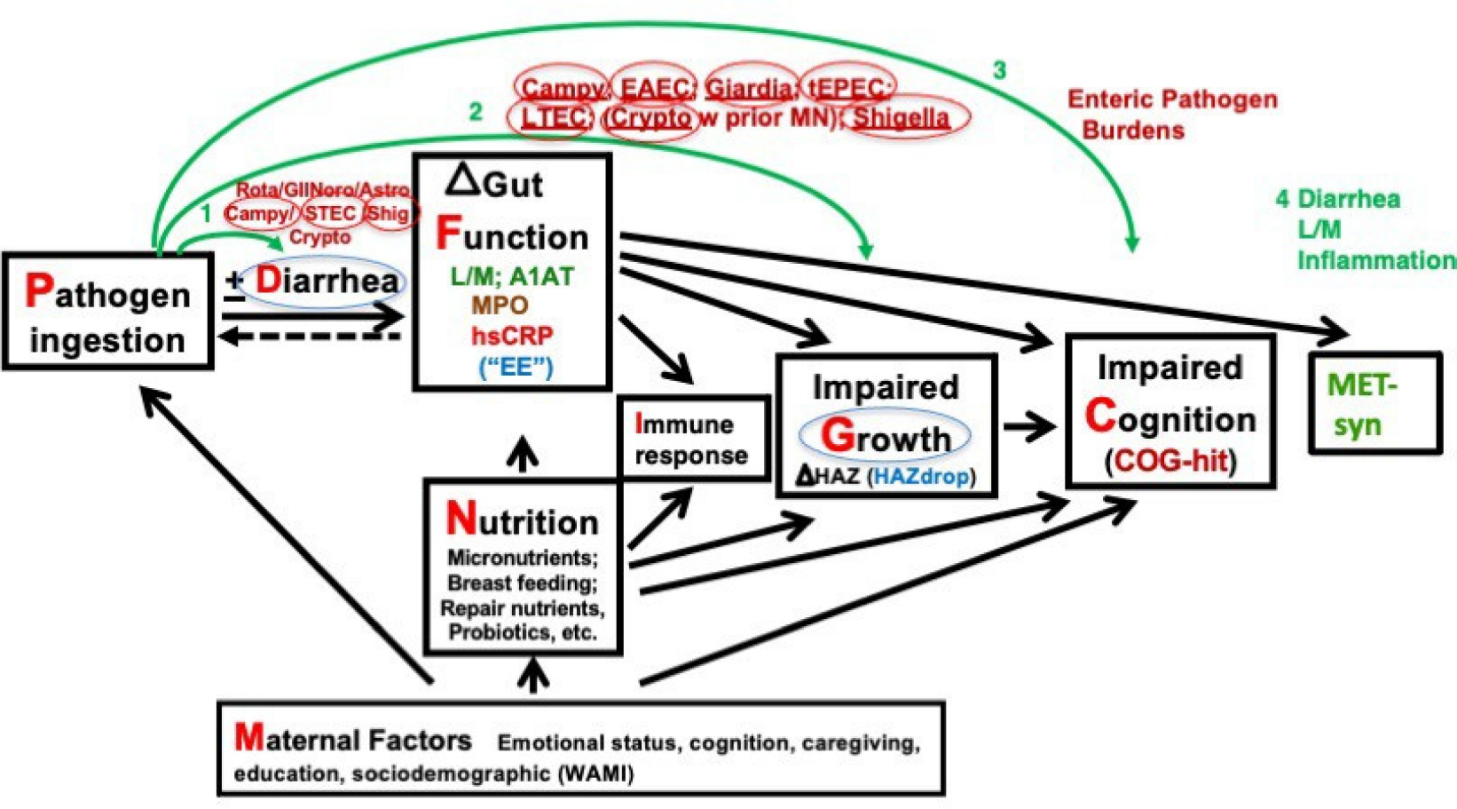
Overview of the major multisite GEMS and MAL-ED studies of enteric pathogen associations with diarrhea or enteropathy, altered gut function, impaired growth, and cognition and metabolic changes. These studies involved over 9000 and 2000 children, respectively.^18,19^ Pathogens marked with red circles have murine models for diarrhea or enteropathy without overt diarrhea as noted in blue circles. L/M = lactulose/mannitol excretion ratio; A1AT = alpha-1-antitrypsin; EE = environmental enteropathy; Campy = *Campylobacter*; Crypto = *Cryptosporidium*; deltaHAZ = change in height-for-age Z score from 4 to 24 months of age; HAZdrop = decline in HAZ scores between 4 and 24 months of age; COGhit = decline in cognitive function (often measured 3–7 years later) associated with enteric infections in the first 2 years of life; METsyn = metabolic syndrome later in life that is associated with early childhood enteric infections; WAMI = water/sanitation, assets, maternal education, income.

Since the seminal studies of Mata, numerous prospective studies showing associations of diarrhea and enteric infections with stunting have been conducted in diverse areas such as Peru,^13,15^ Brazil,^12,16^ Bangladesh,^14^ and elsewhere.^17^ The Global Enteric Multicenter case-control study (GEMS) of moderate–severe diarrhea in 9439 children <5 years old in four African and three Asian sites showed the top five pathogens (by “attributable fraction”) to be rotavirus, *Cryptosporidium*, ST-ETEC, *Shigella*, and “typical” EPEC,^18^ with qPCR extending these findings to include far more *Shigella* infections than previously recognized (including many without bloody diarrhea) and *Campylobacter*, as well as sapoviruses, adenoviruses, and rotaviruses.^11^ In addition, the Malnutrition as an Enteric Disease (MAL-ED) prospective cohort study of over 2000 children at eight sites in Asia, Africa, and South America found enteric infections to be extremely common, often with multiple pathogens in most of the children throughout their first 2 years of life. These pathogens were often detected in the absence of overt diarrhea. These findings have been made possible by the qPCR diagnostics for 29 viral, bacterial, and parasitic pathogens that were performed on over 35,000 monthly fecal specimens (over 7000 with diarrhea and over 26,000 in monthly specimens taken from children without diarrhea). Furthermore, the associations of pathogens with growth failure was seen in the children who did not have overt diarrhea. The bacterial and protozoal infections that were associated with impaired growth were led by *Shigella*, EAEC, *Campylobacter*, and *Giardia*.^10^ These studies and their potential impact are shown in Figure 2.

Regarding specific major infections, for example, of 7601 diarrheal and 26,267 surveillance stool samples, 85% of all 1892 children at the eight MAL-ED sites had *Campylobacter* infections in their first 1 year of life, with significant associations with bloody diarrhea, growth failure (−0.6 HAZ by 2 years old), and with increased fecal myeloperoxidase (MPO), alpha-1-antitrypsin, and serum alpha-1-acid glycoprotein (AGP), suggesting intestinal and systemic inflammatory responses.^20^ Similarly, 95% of these children across all eight MAL-ED sites had EAEC infections, with associations again with fecal MPO and growth failure.^21^ Even *Giardia* infections were seen in 96% of these children and, when seen in the first 6 months of life, were independently associated with subsequent growth failure, much as had also been seen in another study population in Bangladesh.^22,23^

## Potential “Triple Burden” of Enteric Infections

**Figure 3:**
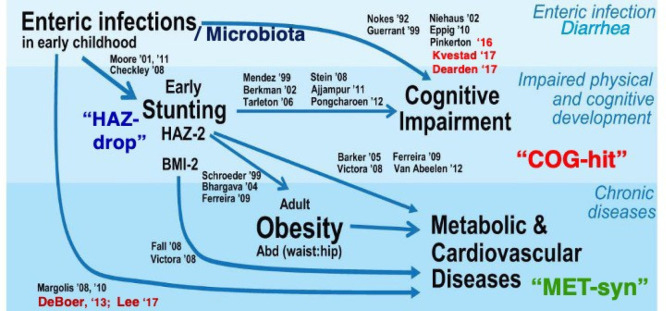
Evidence from many studies showing a potential “triple burden” of common early childhood enteric infections directly or indirectly on diarrhea, growth failure (HAZdrop), cognitive development (COGhit), and potentially lasting metabolic consequences or metabolic syndrome (METsyn).^24−29^ References shown in red are those published since the 2013 reference cited in (24).

Figure 3 summarizes the potential additional lasting “triple burden” of the common and repeated enteric infections in early childhood over time into adulthood. Published data supporting each of these arrows are noted in black and are listed in Guerrant, Nature Reviews, and Gastro Hepatol.^24^ Noted in red are later references that include Pinkerton, 2016; Kvestadt, 2015; Dearden, 2017; DeBoer–Stein, 2013; and Lee–Kosek, 2017.^25−29^

## Models to Explore Roles and Causality

**Figure 4:**
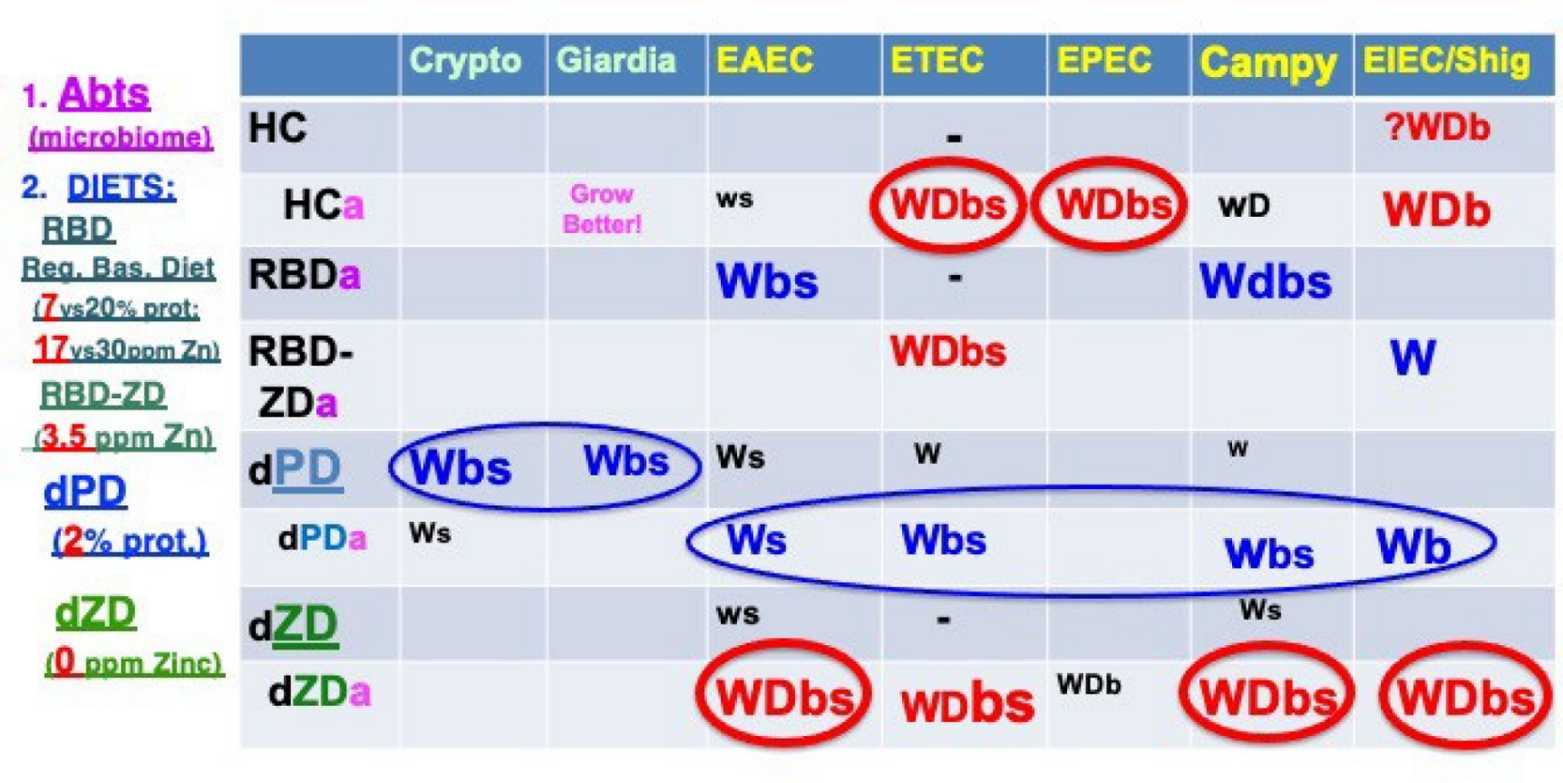
Murine models of enteropathy or diarrhea with their major recognized protozoal and bacterial enteric pathogens. The three key variables shown are (1) antibiotic pretreatment (Abts or a); (2) diet (HC = house chow or normal, with 30 ppm zinc and 20% protein; RBD = regional basic diet with 17 ppm zinc and 7% protein; RBD-ZD = RBD without added zinc, *i.e.*, with 3.5 ppm zinc; dPD = defined protein deficient with 2% protein); and dZD-defined zinc deficient with zero ppm zinc); and (3) pathogen (the protozoa, *Cryptosporidium* and *Giardia* are shown in light green; the bacteria, enteroaggregative *E. coli*, enterotoxigenic *E. coli*, *Campylobacter*, and *Shigella* are shown in yellow). W = weight impairment; D = diarrhea; b = biomarkers of inflammation or epithelial damage; s = shedding; with larger size and circles indicating predominant effects on growth (shown in blue) or diarrhea (shown in red). See references (9−14) and references therein.

As summarized in Figure 4, three elements are key to enteropathy or overt diarrhea outcomes in our murine models of enteric infections: (1) microbiome, (2) host diet, and (3) pathogen. Each element has specificity in its effects on outcomes that range from acute bloody diarrhea to more prolonged enteropathy and growth impairment. In addition to raising potential mechanistic pathways and hypotheses for testing in field studies, these models help open and assess novel interventions and their expected benefits for diarrhea or enteropathy, be it with vaccines, specific nutrients or micronutrients, antimicrobial or other drugs, or selected protective microbiota.

## Roles of the Microbiome

First, regarding the microbiome, as has been shown since the 1950–1960s studies by Bohnhoff, Miller and Martin,^30^ and Hentges^31^ and many others, murine models of bacterial infections require the resident microbiota to be perturbed by antimicrobial treatment to get robust infections. Initial studies used streptomycin (along with antimotility agents) to get robust *Salmonella* infections, and antimicrobial disruption of the resident microbiota is typically required to increase susceptibility to *Clostridioides difficile* infections in humans as well as in murine models. This is further evident by the exquisite sensitivity of gnotobiotic mice to enteric infections.^32^ However, while antibiotic treatment is key to getting robust *initial* infections with bacterial pathogens, it has minimal effects against protozoal infections such as cryptosporidial infections and even protective effects against *Giardia* infections.^33,34^

## Roles of Dietary Protein or Zinc

**Figure 5:**
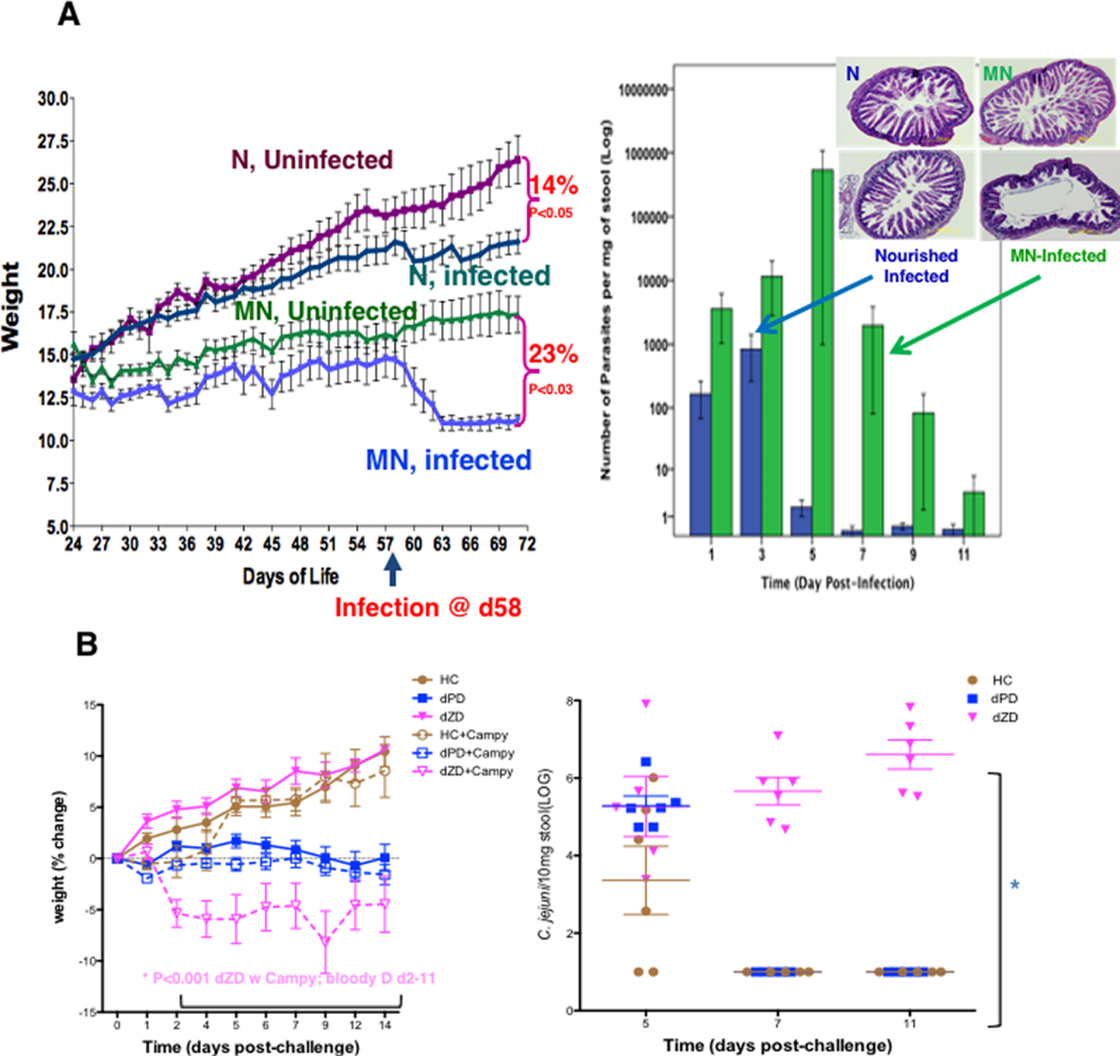
(A) Cryptosporidium causes and worsens malnutrition. Cryptosporidial infections with unexcysted oocysts (infected) in weaned C57Bl/6 mice caused a 14% weight decrement over 2 weeks in normally nourished (N) mice, while malnourished (MN) mice (on defined protein deficient, dPD diet) had an additional 23% decrement in their weight over 2 weeks after infection. Furthermore, malnourished mice shed up to 5 logs more Cryptosporidial parasites than nourished mice given the same inoculum at the same time, with similar striking greater histologic damage with combined infection and malnutrition.^34,35^ (B) Zinc deficiency enables prolonged Campylobacter shedding, weight loss, and bloody diarrhea.^37^

During initial attempts to obtain robust infections in mice that resulted in reductions in weight gains that mimic the growth impairment seen with *Cryptosporidium* infections in children in developing areas, it was quickly found that protein deficiency (even when kept isocaloric) dramatically increased the weight decrements as well as the intensity of infection by *Cryptosporidium parvum* oocysts. As shown in Figure 5A, the combined effects of protein deficiency with cryptosporidial infection were dramatically greater than either in isolation on weight, intestinal damage, and on parasite shedding (the latter by up to 5 logs greater than matched infections without protein deficiency).^34,35^ Furthermore, the likely basis for the enhanced severity of cryptosporidial infection is the delayed epithelial cell turnover with protein deficiency that delays both apoptotic clearance of infected intestinal epithelial cells as well as a delay in their replacement by proliferative crypt cells that are typically renewing the intestinal epithelium every 3–5 days in mice as well as humans.^36^

The other dietary determinant that we have found to profoundly affect the “clinical” as well as microbial outcomes of enteric infections is zinc deficiency (or treatment). As with the microbiome, the impact of zinc availability varies with each pathogen, likely because of increasingly documented effects of zinc on the expression of not only host but also microbial traits. Again, many have elucidated the myriad effects of zinc on metabolic pathways as well as on gene expression. Our experience has shown that zinc deficiency increases two interesting host genes in our murine model of EAEC infections (but only in the presence of EAEC), namely, *MUC2* and *CFTR*.

Numerous studies have documented the importance of zinc for the healthy growth, immunocompetence, and neurobehavioral development in children.^38^ It is estimated that 17% of the world’s population is at risk of inadequate zinc intake, and this risk is correlated with stunting prevalence.^39^ In addition, inflammation has been associated with low zinc levels (as we have also seen with zinc deficiency in our murine models; see below) but with higher serum ferritin and soluble transferrin receptor (sTR) in children.^40^ Zinc levels may also be low in association with enteropathy, perhaps from reduced absorption, but, fortunately, oral zinc is still effective in restoring zinc levels even in children with evidence of enteropathy.^41^

Finally, adjustments in serum zinc measurements for systemic inflammation, though suggested by some, seems inconsistent and often not necessary in interpreting zinc levels in children with or without simultaneous evidence of systemic inflammatory responses (as by CRP or AGP).^42^

We have documented the serum and tissue zinc levels seen in our murine models after 2 weeks of conventional, limited zinc, or no zinc diets for comparison with zinc concentrations considered to be normal or low in children. The serum levels of zinc in our mice after 2 weeks on house chow or “defined nourished” diets (each with 30 ppm zinc) and even those on the regional basic diet (RBD) with 17 ppm zinc were all tightly within the middle (*i.e.*, 0.8 to 0.95 μg/mL) of the normal range of zinc levels in children (0.7–1.1 μg/mL). Mice given the defined nourished diet containing no measurable zinc for 2 weeks had very low serum zinc levels of only 0.2 to 0.4 μg/mL. Those on a limited zinc diet (RBD with no added zinc; RBD-ZD with 3.2 ppm zinc) had moderately low levels of zinc ranging from 0.5 to 0.7 mg/mL. In sum, defined fully nourished (N) and regional basic (RBD) diets, with zinc in the diets of 30 and 17 ppm zinc, respectively, showed similar serum zinc levels in our mice that were in the middle range of normal for children. However, the serum zinc levels in mice after 2 weeks on a defined diet that had no measurable zinc were very low, substantially below the lowest normal range for children. In comparison, the RBD-ZD diet had serum zinc levels that were comparable to or just below the normal range for children. Indeed, these span the ranges of zinc concentrations that have varied effects on different pathogen replication and virulence trait expression. For example, even these mild to moderate zinc-deficient levels (in comparison to those seen with severe zinc deficiency) were sufficient to reduce selected virulence traits as well as phenotype (such as biofilm) expression, both *in vitro* and *in vivo*. In our published models of EAEC, ETEC, *Shigella*, and *Campylobacter* infections, zinc deficiency and zinc treatment often have profound effects. For example, not only host gene expression but also histopathologic damage and selected (aggR-dependent) EAEC virulence trait expression are all increased in infected mice on the severe zinc-deficient diet.^43−45^ Even physiologic levels of zinc (*i.e*., 0.01 mM) dramatically suppress the EAEC virulence trait expression *in vivo*. Furthermore, zinc deficiency profoundly alters weight loss, pathogen shedding, and biomarkers of intestinal disruption in EAEC, ETEC, *Shigella*, and *Campylobacter* infections in our antibiotic treated C57Bl/6 weaned mouse models.^37,45−48^ Despite the many excellent field studies of zinc supplementation mentioned above, it is difficult to incorporate pathogen- or virulence-trait-specific effects in field studies. However, our murine models can enable study of zinc supplementation on pathogen- and virulence-trait-specific responses. Specifically, we have found that zinc treatment reduces inflammation with EAEC or *Shigella* infections and selectively alters much, but not all (*i.e.*, not *eltA*), expression at zinc levels that are “subinhibitory” for bacterial growth. Further study of specific effects of zinc on pathogen virulence trait expression are currently underway in our laboratory.

## Roles of Specific Pathogens

A recent review of models of EE and malnutrition has emphasized the importance of diet and microbial components, especially in severe acute malnutrition (SAM) with or without specific infections.^49^ However, the frequency of even multiple “asymptomatic” enteric pathogen infections that are now recognized with quantitative molecular diagnostics, as well as the association of these asymptomatic pathogen burdens in early childhood with growth failure over several years, warrant careful assessment of selective and broader interventions as well as their limitations.^10,11^ In addition, antibiotic use is extremely common in children living in impoverished areas at risk of heavy and repeated enteric infections.^50^ Our murine models with the different effects of protein- or zinc-deficient diets as well as previous antimicrobial agents to alter the microbiome and susceptibility are consistent with the major currently recognized bacterial and protozoal enteric infections associated with growth failure in children across multiple sites in Asia, Africa, and South America. Most importantly, these models enable the dissection of potentially remediable dietary and pathogen components in ameliorating the outcome of malnutrition and enteric infections and their outcomes for child growth and development. Furthermore, biomarkers to track critical determinants of these adverse outcomes, as well as their potential mechanisms and remediation, can also be assessed. Examples include readily measurable fecal markers such as MPO for inflammation and lipocalin-2 (LPO), A1AT, or even systemic bloodstream markers of intestinal damage, disrupted gut permeability, and systemic absorption seen with drivers of inflammation such as LPS. Even the metabolomic consequences of each dietary component, pathogen, and intervention can also be assessed in these relatively inexpensive murine models for comparison with metabolomic effects in affected children in the field.^51,52^ Protein- and zinc-deficient diets modulate the murine microbiome and metabolic phenotype.^37,51^

As an example of one of the leading bacterial pathogens, Figure 5B summarizes our published murine model of *Campylobacter* infections. Also published are the details of our models of ETEC, *Shigella*, EAEC ,and EPEC infections over the past decade. Each of these top enteropathy and diarrheal pathogens shows clear and distinctive differences in their dependency on protein or zinc deficiencies in the diet. All of the bacterial pathogens, but not the protozoal pathogens, *Cryptosporidium* or *Giardia*, require alteration of the normal microbiota to get a robust model of infection. Strikingly, *Giardia* is just the opposite, having a greater effect on growth and other outcomes only when normal or pathogenic microbiota are left intact (*i.e.*, without prior antibiotics).

Even with antibiotics given before infection, however, a robust, lasting infection with *Campylobacter* beyond 5 days is totally dependent on zinc deficiency. Similarly, the severity of both weight loss and of diarrhea that is often bloody (as seen in malnourished children) is much more severe and lasting in the zinc-deficient mice, compared with normally nourished mice, as shown in Figure 5B. Not shown, we have extended these findings to develop a milder model of enteropathy with the reduced zinc RBD diet that has been helpful in studying several novel enteropathy interventions, such as vaccines, novel therapeutics, or inexpensively produced immunotherapeutics, nutrient therapeutics, micronutrients, and probiotics.

We have also described the effects of protein- or zinc-deficient diets on weight decrements, fecal pathogen shedding, overt diarrhea, and fecal biomarkers of inflammation with heat labile + heat stable enterotoxigenic *E. coli* (LT+ST ETEC). In contrast to *Campylobacter* infections, however, ETEC infections actually had their greatest effects on weight decrements and on shedding in mice on the full house chow normal diet. However, the effect of ETEC on intestinal inflammation or disruption by fecal MPO and lipocalin-2 was progressively worse in mice on protein- or zinc-deficient diets. Even protein or zinc deficiency alone was associated with increased MPO and LCN, albeit even more so when also infected with ETEC. Subtle differences in the apparent zinc dependency or suppression of ST or LT expression, respectively, are also detailed in the paper by Bolick in 2018.^46^

Intermediate between *Campylobacter* and ETEC infections, *Shigella* infections show the greatest effect on acute weight loss and diarrhea over 1–3 days after infection, but these clinical effects, like fecal pathogen shedding, are transient unless the mice are zinc-deficient.^53,54^

In further contrast to *Campylobacter*, ETEC, or *Shigella* infections, EAEC and *Cryptosporidium* infections were made worse by protein deficiency but not zinc deficiency. In addition, zinc supplements that inhibited both biofilm formation by EAEC *in vitro* also reduced and mouse intestinal colonization by EAEC.^44,47^ Most recently, we have also published that EPEC can also cause diarrhea and intestinal inflammation in our antibiotic-treated C57Bl/6 mouse model.^55^

## Can Murine Models Enable Relevant Work on COGhit and METsyn as Well as Diarrhea and HAZdrop?

### Modeling Synaptogenesis (Relevant to Hippocampal COGhit)

While testing higher executive function or semantic fluency seen in older children is difficult, if not impossible to model in mice, murine models do indeed offer unique opportunities to study morphological/physiological and behavioral alterations induced by malnutrition or EE. It has been shown that altered hippocampal architecture and synaptogenesis and even responses to nutrient/micronutrient therapy can be seen with malnutrition in mice.^56^ These findings appear to have potential relevance to similar treatment effects in children in developing areas.^57^

## Metabolomics to Help Model METsyn

The recent emergence of high-throughput tools that can comprehensively assess the developing metabolome and microbiome of the experimental models and children in these settings provides a means to characterize the potential metabolic consequences (METsyn) of these insults. Metabolomics and metataxonomics/metagenomics are system biology techniques that can agnostically capture a huge amount of metabolic and/or microbial information in a single measurement, respectively. This unbiased approach avoids the need for predefined hypotheses based on existing knowledge allowing novel insights to be gleaned. The application of metabolomics to biofluid (urine, plasma, and fecal water) and tissue samples from murine models of malnutrition and infections has identified a wide range of metabolic perturbations to be induced by these different insults.^51,58,59^ Such disruptions are specific to nutritional deficiencies, infections, and their combinations. This includes a range of biochemical pathways including those involved in energy, amino acid, choline, niacin/nicotinamide, and nucleic acid metabolism. Importantly, many of these metabolic derangements are consistent between the murine models and children studied.

An age-dependent maturation of the microbiome and metabolome has been identified in children that can be delayed by malnutrition.^60,61^ Such microbial and biochemical maturation is important to support the physiological, immunological, biochemical, and microbiological needs of the developing infant. Understanding and measuring this maturation process and the status of individual infants will be important to inform and direct novel targeted interventions. Furthermore, modulation of the developing microbial and metabolic system in early life may predispose individuals to adverse outcomes in later life, such as metabolic syndrome and cardiovascular disease. Combining these two complementary techniques of analyzing both the microbiome and the host metabolic state can enhance our understanding of the microbial and metabolic effects of malnourishing diets and infections and their interactions. For example, in studying the combined metabolomic and microbiome effects of cryptosporidial infections,^62^ we can distinguish the metabolites that associate with microbial alterations from those associated with host metabolism. Indeed murine *Cryptosporidium* infections were associated with host metabolite trimethylamine oxide that has been associated with risk of death from cardiovascular diseases and strokes in humans.^52,59,63^ Such metabolic consequences in murine models of enteric infections enable further study of mechanisms and pathways involved and potentially relevant interventions.

## Modeling Coinfection Interactions

Finally, murine models of specific pathogen infections enable assessments of the potential roles of combined infections that may help us better understand such complex interactions as *Giardia* and enteroaggregative *E. coli* (EAEC) infections.^64,65^ These findings suggest that when interacting with such bacteria as EAEC or even certain common resident microbiota, *Giardia* can have detrimental effects on weight, enteropathy, and metabolism, while without EAEC (or following broad spectrum antimicrobials), *Giardia* may have opposite effects, much as has been seen in conflicting clinical studies.

## Examples of Applying These Murine Models to Assess Potential Interventions

While the ultimate predictive value of using these models described herein will be determined only when promising findings are tested in human studies, we have been impressed that many of the clinical manifestations seen in children can indeed be seen in these murine models. We have applied our models of *Shigella* and ETEC infections in a promising candidate multi-pathogen live attenuated vaccine in collaboration with Dr. Eileen Barry at the University of Maryland.^53^ This demonstrated protective efficacy when tested with the identical immunization protocol followed by either *Shigella* or ETEC challenge, hence, suggesting that clinical studies could well consider either infection in children as potential outcomes of interest. In addition, we have shown similar proof of concept with passive protection against ETEC diarrhea when the inoculum was mixed with a specific monoclonal secretory antibody (SIgA2 anti-ETEC CfaE) produced in plants.^66^ Several other promising passive antibodies given either at the time of or even after established infections have shown promise and are currently under study and in preparation for publication, and additional active multi-pathogen vaccines are under collaborative study in our murine models.

## Synthesis and Conclusions

**Figure 6:**
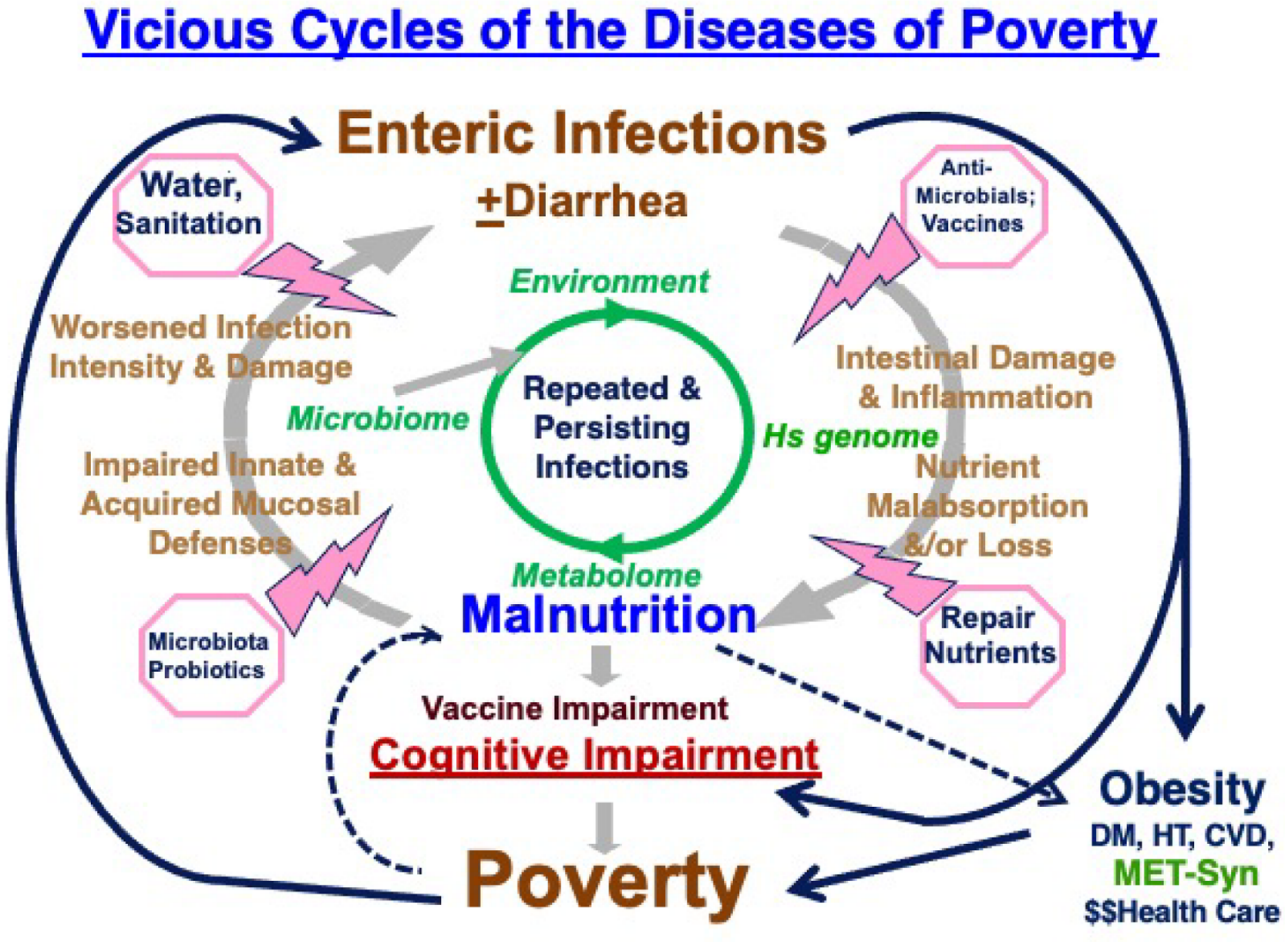
Vicious cycles of enteric diseases of poverty, showing outcomes, pathways, and potential sites for interventions.^24,68,69^ Potential interventions are suggested in pink; potential mechanisms are shown in light tan, and environment–host–metabolic–microbiome variables are shown in green. DM = diabetes mellitus; HT = hypertension; CVD = cardiovascular disease.

In conclusion, enteric infections still kill over a thousand children each day (nearly 0.5 million/year) and also contribute to moderate to severe stunting of over 160 million children who do not die, but whose full growth and development are impaired in impoverished areas. Enteric infections, in combination with malnutrition, also increasingly appear to impair the cognitive development of millions of children living with repeated, multiple, and persistent infections in their most formative early childhood when critical brain synaptogenesis occurs. Finally, enteric infections and environmental enteropathy in early life may predispose to later life obesity, metabolic syndrome, and costly lifelong consequences. Collectively, these often hidden or “silent” long-term consequences of stunting (HAZdrop), cognitive impairment (COGhit), and metabolic syndrome (METsyn) make early childhood enteric infections, malnutrition, and environmental enteropathy indeed both drivers and consequences of poverty itself, in a vicious cycle, as shown in Figure 6.^67^

Thus, models that help us dissect the key components of the causes and consequences of enteric infections and environmental enteropathy become critical to our understanding of their improved recognition and amelioration that are so important to over 160 million of the world’s children living in poverty. Promising molecular and metabolomic tools can help us dissect mechanisms involved and develop biomarkers and interventions to control these infections and enteropathy and their potentially devastating consequences worldwide.
